# How Does Secure-Base Leadership Affect Employees’ Taking-Charge Behavior: The Role of Psychological Availability and Independent Self-Construal

**DOI:** 10.3390/bs13100853

**Published:** 2023-10-18

**Authors:** Huanhuan Li, Yanbo Zhang

**Affiliations:** School of Management Engineering & Business, Hebei University of Engineering, Handan 056038, China; 17698062597@163.com

**Keywords:** secure-base leadership, taking-charge behavior, psychological availability, independent self-construal

## Abstract

Under the VUCA background, enterprises need to actively change to meet the requirements of internal and external environmental changes. This article surveyed 250 employees. Using statistical software SPSS 27.0 and Process 4.1, we address the outcome of secure-base leadership on employees’ taking-charge behavior by considering the influencing mechanisms and boundary conditions. The results indicate that secure-base leadership positively shapes employees’ taking-charge behavior, and psychological availability plays a mediating role in the relationship between secure-base leadership and such employee behavior. Independent self-construal positively moderates the impact of psychological availability on employees’ taking-charge behavior and positively moderates the indirect impact of secure-base leadership on such behavior through psychological availability. Our findings could enrich the empirical research on employees’ taking-charge behavior by secure-base leadership, thereby promoting the sustainable development of organizations.

## 1. Introduction

With the rapid development of digital information technology, organizations are currently in a VUCA (volatility, uncertainty, complexity, and ambiguity) environment. As such, changes in organizational structure, management style, and so forth, have become a means of coping with future development, which not only requires companies to make changes in all aspects of their macro-strategies, but also encourages employees of the organization to take initiatives to change work methods [1]. However, taking-charge behavior may be understood as challenging the status quo and carry negative consequences for employees, causing them to maintain a cautious attitude toward such behavior [2]. Therefore, how to stimulate employees’ taking-charge behavior is a problem that needs to be solved by both academia and corporate organizational change.

In organizational contexts, employees and leaders interact closely and frequently every day, and due to the asymmetry of status and power between leaders and employees, leaders’ behavior significantly affects employees’ cognition, attitude, and behavior [3]. So, what kind of leadership behavior can more effectively motivate employees’ taking-charge behavior? Taking-charge behavior is a spontaneous and constructive effort by employees to change the way they work in order to achieve functional change in the organization [4]. In recent decades, there have been incidences of employee’ taking-charge behavior, including general self-efficacy [4,5], employee psychological empowerment [6], leadership styles [7,8], prosocial motivation [9], supervisor developmental feedback [10], and superior-subordinate relationships [11]. Among several possible influential factors, leadership has been identified as a primary driver of employees’ taking-charge behavior [12]. As such, empowering leadership [7], self-sacrificial leadership [8], inclusive leadership [13], green transformational leadership [14], and shared leadership [15] have been proven to affect employee taking-charge behavior. Uhl-Bien has called for further research on relational leadership—that is, changing economic conditions that requires leaders to be more attentive to relationship-building to create a more motivated workforce [16]. As a relationship-oriented leadership style, secure-base leadership is a new leadership style that has emerged in the field of organizational behavior research [17]. This leadership style recognizes the value of employees, indiscriminately accepts others, integrates care and challenges, and establishes emotional bonds with employees and provides them with “secure-base” support. On the one hand, this style may enhance employees’ sense of protection and eliminate worries when they arrive at new ideas; on the other hand, by encouraging exploration and adventure, it stimulates employees’ creative thinking [18]. Therefore, this article attempts to explore the impact of secure-base leadership on employees’ taking-charge behavior in the context of China.

Secure-base leadership can promote employees’ proactive behavior by influencing their self-efficacy [19], while also having a certain impact on their innovative [20], voice [21], and helpful behaviors [22]. The research on secure-base leadership is in its infancy, and its outcomes attract trivial attention [17]. The mechanism by which secure-base leadership encourages employees to challenge difficulties and influence their taking-charge behavior has not been fully explored. A secure-base leader can establish a “interpersonal bond” with employees, making them feel trusted and encouraging them to propose suggestions or ideas beneficial to the organization to repay the leader and the organization. It can also establish a “goal bond” with employees, providing them with the determination and resilience to face risks and overcome difficulties, which coincides with the essence of employees’ taking-charge behavior [23]. Taking-charge behavior is full of uncertainty and risk. Generally, employees are reluctant to initiate such behavior without authorization from leaders or organizations. In a highly competitive environment, taking-charge behavior is more helpful than the passive change in seizing opportunities [24]. So, the foremost point is how to promote taking-charge awareness and whether secure- base leadership could effectively promote such behavior. At present, the academic community has not provided an answer to this research question. However, existing research has not directly examined the relationship between secure-base leadership and employee’ taking-charge behavior.

As a precursor to stimulating a series of specific behaviors among employees, psychological availability is an important factor that reflects employees’ psychological cognition, reflecting the degree to which they can respond to their physiological, emotional, and cognitive needs at work [25]. Employees with high psychological availability have stronger inner resources, greater creativity, and perseverance. Such employees and are able to learn, explore, and find new paths, thus participate more deeply and actively in creative and innovative work [26]. Previous research has confirmed the effects of various leadership styles on psychological availability, such as emergent leadership [27], inclusive leadership [28], and empowering leadership [29], but there has not been any research on psychological availability by secure-base leadership. We chose psychological availability to explore the internal mechanism process between secure-base leadership and employees’ taking-charge behavior. Secure-base leaders tend to listen, ask questions, and solve problems through equal dialogue and communication with employees. This helps to eliminate power barriers within the organization and enhance employees’ psychological safety [30]. Such leaders also provide clear opportunities for employees to actively explore and innovate generate positive psychological implications. They also provide employees with a “dare to be the first” psychological environment [20], stimulate employees’ usable perceptions of their own physiological, emotional, and cognitive resources, and meet their psychological availability. When an individual has a high level of psychological availability, it indicates that they are fully prepared for the physical, emotional, and cognitive requirements of their work [31]. This availability strengthens employees’ work engagement and helps them maintains a positive and explorative work state, which may promote taking-charge behavior [32]. Therefore, this article attempts to introduce psychological availability to better explore the impact of secure-base leadership on employees’ taking-charge behavior.

Individuals with independent self-construal emphasize progress of the self [33]. In organizations, individuals with high independent self-construal value their inner thoughts, emotions, and behaviors, and their response to situations is based on how to express their own internal qualities and characteristics [34]. Compared to employees with low independent self-construal, employees with high independent self-construal are less worried about their leaders resisting their change behavior, lack a strong sense of anxiety about taking-charge behavior, and are willing to demonstrate their abilities without concealment. Thus, such employees are more willing to engage in take-charge behavior [35]. This article introduces independent self-construal as a moderating variable to further explore the internal mechanism of secure-base leadership on employees’ taking-charge behavior, and ultimately establish a moderated mediation model.

## 2. Theoretical Basis and Hypotheses Development

### 2.1. Secure-Base Leadership and Taking-Charge Behavior

The concept of a secure-base emerged out of Bowlby’s research on attachment theory, which was originally used to describe the relationship between mothers and infants. Bowlby essentially defines a secure-base as behavior that responds, encourages, or assists the other person whenever they need it, but actively intervenes only when the need is obvious [30]. Scholars have since expanded the secure-base into the field of leadership studies, arguing that secure-base leaders give their employees the inspiration and energy they need to take on challenges by on providing care [18]. Secure-base leadership respects employees, recognizes their contributions, provides necessary help to their growth [13], and could effectively empower employees and encourage them to participate in decision making. Some scholars believe that secure-base leadership encourages employees to realize their potential, take ownership of their goals, and attempt new ideas, as well. Such leadership is helpful to employees under times of duress and in positive problem solving [19]. Subsequently, scholars at home and abroad have studied the impact of secure-base leadership on specific employee behaviors, but no research currently exists on secure-base leadership regarding employees’ taking-charge behavior.

Taking-charge behavior is a behavior that requires individual employees to make organizational functional changes in the way work is performed in their unit or organization through a voluntary and constructive effort [4]. Secure-base leaders excel at stimulating employees’ potential and enhancing their confidence. They also carry out effective authorization to make employees feel they have more control over resources, thereby inspiring them to proactively identify problems in the organization and actively change to promote its development [20]. First, when employees encounter difficulties, secure-base leaders provide employees’ with support and assistance and try to solve their problems, thereby narrowing the distance between leaders and employees [23]. Such leaders encourage employees to open up and point out problems existing in their organizations, and provide employees with open communication channels, increasing their trust in their leaders. If employees do not achieve the expected results, they will reduce their fear of punishment [36]. Second, when employees want to improve in their work, secure-base leaders can encourage them to pursue personal goals and achieve self-development, which provides employees with the space to grow and enhances their sense of competence [37]. Finally, when employees find problems in their organization but do not express them, secure-base leaders will delegate certain powers to subordinates and effectively authorize them [22], which gives employees a sense of belonging. This enables employees to engage in taking-charge behavior and do their best for the development of the organization. As a result, we present the following hypothesis:

**H1:**  *Secure-base leadership has a significant positive effect on employees’ taking-charge behavior*.

### 2.2. The Mediating Role of Psychological Availability

Psychological availability is the feeling of availability of an individual’s own physical, emotional, or psychological resources at a given moment in time, which has an impact on the person’s attitudes and behaviors [25]. In subsequent research, scholars have expanded psychological availability to the field of organizational behavior, arguing that psychological availability defines an individual’s state of readiness. When engaged in the workplace, employees with high psychological availability are able to readily invest their acquired physical, emotional, and cognitive resources into their work roles, thus demonstrating higher levels of commitment [38]. First, the availability characteristics of secure-base leaders conveys to individuals that they can grasp the signals of the external environment, and that as leader and the object of their attachment, is able to assist in overcoming the difficulties of the process by providing timely help. Employees in such an environment will greatly increase their ability to perform with confidence, perception of competence, and improved emotional needs [22]. Second, secure-base leadership includes encouragement of growth characteristics, which can encourage employees to grow and care about their own physical and mental health. Employees who perceive this encouragement and care can gain psychological stability, increase their sense of identity with the organization, and gain confidence in acquiring resources. In addition, their perception of the availability of physiological, emotional, and cognitive resources will be strengthened [22]. Finally, the noninterference characteristic of secure-base leadership can provide employees with autonomy, which improves their control over the work environment to a certain extent and gives them the opportunity to enhance their knowledge and skills, which improves their perception of cognitive resources [39]. In addition, secure-base leaders care about employees and are prone to listen to their opinions. This will make employees feel respected by the organization, strengthen their psychological empowerment [40], and promote taking-charge behavior among employees. As a result, we present the following proposed hypothesis:

**H2:**  *Secure-base leadership has a significant positive effect on psychological availability*.

When employees feel that their leaders care about them, their psychological availability is fully stimulated, and they perceive abundant available resources, thus becoming confident in addressing changes in the workplace and successfully completing work tasks [41]. Organizations featuring fairness, tolerance, and trust are conducive to enhancing employees’ psychological safety, and spur them to devote themselves to personal learning and abilities, thereby facilitating the use of new knowledge and methods in their work [13]. First, confident employees are more inclined to change the current situation to adapt to changes in the external environment. They actively identify potential problems in the organization, and even in the face of setbacks, they remain calm and develop corresponding strategies to solve difficulties in the organization [42]. Second, since individual behavior is affected by the situation and individual values, secure-base leadership provides employees with a good working atmosphere and available resources, and makes employees feel the warmth of the organization [23], thus improving their emotional and cognitive needs. Finally, secure-base leadership has the characteristic of noninterference to employees, which makes employees cope with the challenges brought by organizational changes. Therefore, the caring and challenging characteristics exhibited by secure-base leaders can help employees eliminate inherent threats in the environment, thus increasing their psychological safety [43], and improving their psychological availability. Employees with high levels of psychological availability will have their physiological and psychological needs meet, which may promote taking-charge behavior. As a result, this paper presents the following proposed hypothesis:

**H3:**  *Psychological availability mediates the relationship between secure-base leadership and employees’ taking-charge behavior*.

### 2.3. The Moderating Role of the Independent Self-Construal

Self-construal, first proposed by Markus and Kitayama, refers to the tendency of individuals to perceive themselves in terms of the frame of reference in which they place themselves. The authors argued that different cultures influence people to form different types of self-construal—independent self-construal and dependent self-construal [44]. Individuals with high independent self-construal are inclined to receive appraisals that are beneficial to themselves. And even if obtaining negative feedback, they show strong self-protection tendencies. Therefore, individuals with high independent self-construal are more confident and believe in their ability to cope with organizational change [45].

When employees’ psychological availability is satisfied, individuals with high independent self-construal will produce a perception of their own value in the organization, feel valued by the organization [46], and show their self-opinion more actively. They also they regard themselves as a part of the organization, and hope that it will develop in a better direction, and as such, they will find organizational problems in time and take the initiative to adopt change behaviors. First, high independent self-construal employees have strong confidence in themselves [47] and believe that they can meet the physical, ability, and emotional needs required for their work. Their psychological availability is stronger, and they are more inclined to participate in progressive activities to achieve organizational goals [33], thus more likely to engage in taking-charge behavior. Second, when high independent and self-construal employees have a positive evaluation of their influence and value in the organization, their psychological availability is higher and they are more likely to choose to follow their inner thoughts and implement taking-charge behavior [48]. Finally, due to the pursuit of personal satisfaction and sense of achievement by high independent self-construal employees—who are willing to showcase abilities rather than suppress them—their psychological capital is higher [35]. Therefore, they will demonstrate their strength and obtain a sense of achievement by implementing organizational behavior [49]. When they perceive deficiencies in the organization’s workflow or rules and regulations, they are more inclined to adopt a strategy of directly expressing their ideas and implementing actions. As a result, this paper presents the following proposed hypothesis:

**H4:**  *Independent self-construal positively moderates the relationship between psychological availability and employees’ taking-charge behavior. That is, the higher the independent self-construal, the stronger the positive impact of psychological availability on employees’ taking-charge behavior*.

**Figure 1:**
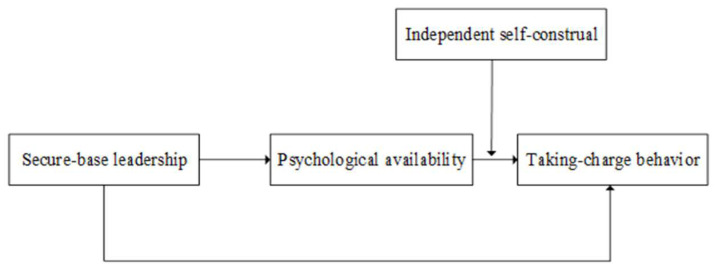
Theoretical model.

Further, because independent self-construal reinforces the impact of psychological availability in employees’ taking-charge behavior, and given that psychological availability acts as a mediator in the relationship between secure-base leadership and employees’ taking-charge behavior, independence self-construal also moderates that mediating effect—i.e., there is a moderated mediation effect (Figure 1). Independent self-construal positively moderates the mediating effect of psychological availability in the relationship between secure-base leadership and employees’ taking-charge behavior. Specifically, the higher the independent self-construal, the stronger the mediating effect of psychological availability. On the contrary, the lower the independent self-construal tendency of employees, the weaker the effect of secure-base leadership on employees’ taking-charge behavior transmitted through psychological availability. As a result, we present the following proposed hypothesis:

**H5:**  *Independent self-construal moderates the mediating effect of psychological availability between secure-base leadership and employees’ taking-charge behavior*.

## 3. Methods

### 3.1. Data Collection and Participants

This study selected five enterprises from Henan and Hebei as samples, covering industries related to communication and finance. Questionnaires were distributed to classmates and colleagues who have worked around the author to collect data via Wen Juan Xing, a specialized online data service company. A total of 265 questionnaires were distributed, out of which 250 were valid, resulting in a valid recovery rate of 94.34%. Table 1 displays the descriptive statistics of the respondents.

### 3.2. Measurements

All the instruments employed in this study were certified in previous studies. To minimize the subjective bias, we adopted Brislin’s translation–back translation procedure when translating the instruments into the Chinese version. All the scales were rated on five-point Likert (higher scores correspond with better performance) without inverted items.

Secure-base leadership scale: this study used a nine-item measurement scale developed by Wu and Parker [19] and included three dimensions: leader availability, encouragement, and noninterference. Some example entries are “My supervisor is sympathetic and supportive when I am worried or upset about something”, “My supervisor gives me encouragement and support when I have a difficult and stressful task or responsibility”, and “My supervisor offers to provide advice or assistance when I need help with a difficult task or problem”. The Cronbach’s alpha coefficient for this scale in this study was 0.900.

Taking-charge behavior scale: This study used a ten-item single dimensional scale developed by Morrison and Phelps [4]. A typical item was “This person often tries to change organizational rules or policies that are nonproductive or counterproductive”. The Cronbach’s alpha coefficient for this scale in this study was 0.927.

Psychological availability scale: This study used a five-item single dimensional scale developed by May, Gilson, and Harter [50]. An example entry was “I am confident in my ability to handle competing demands at work”. The Cronbach’s alpha coefficient for this scale in this study was 0.921.

Independent self-construal scale: This study used a self-construal scale developed by Li and Wei based on Chinese scenarios, with six items related to independent self-construal [51]. An example entry was “It is important for me to have personality traits that are not constrained by others”. The Cronbach’s alpha coefficient for this scale in this study was 0.903.

Control variables: Referring to existing literature research, four variables were selected as the control variables: gender, age, education level, and tenure.

## 4. Data Analysis and Results

### 4.1. Common Method Variance Test

The present study utilized the Harman one-way approach to test the issue of common method bias. An unrotated exploratory factor analysis was conducted together with the topics of secure-base leadership, psychological availability, independent self-construal, and taking-charge behavior. Results showed five factors with eigenvalues greater than 1, and the first factor had a variance of 37.365%. According to Hao and Long, not reaching the 40% level indicates that there is no serious common method bias [52].

### 4.2. Confirmatory Factor Analysis

This study used Amos 24.0 to conduct a confirmatory factor analysis on the study variables. Due to the large number of independent and dependent variable questions, we parceled the independent variables into different dimensions, while also parceling the dependent variables. The results show that the four-factor model (SBL, PA, ISC, TCB) has a better fitting performance (χ^2^/df = 2.845, IFI = 0.936, CFI = 0.935, TLI = 0.921, RMSEA = 0.086) than the three-factor, two-factor, and single-factor model, and thus the four-factor model in this study is the best model (Table 2). This provides evidences for further hypotheses tests.

### 4.3. Descriptive Statistics and Correlation Analysis

This study used SPSS 27.0 to conduct descriptive statistics and a correlation analysis on the variables. The mean, standard deviation, and correlation coefficients of each variable are shown in Table 3. Among them, a significant positive correlation exists between secure-base leadership and taking-charge behavior (r = 0.300, *p* < 0.01), between secure-base leadership and psychological availability (r = 0.260, *p* < 0.01), and between psychological availability and taking-charge behavior (r = 0.821, *p* < 0.01). These results provide preliminary support for the hypotheses tests.

### 4.4. Hypotheses Tests

#### 4.4.1. Main Effect and Mediating Effect Test

This study employ statistical software SPSS 27.0 and Process 4.1 plug-in to test the effect of secure-base leadership on taking-charge behavior and the mediation effect of psychological availability. Gender, age, education level, and tenure were included in the regression model as control variables.

This study tested the mediating effect of psychological availability based on Wen Zhonglin’s stepwise regression method. First, we tested the effect of secure-base leadership on taking-charge behavior. Second, we tested the role of secure-base leadership in influencing psychological availability. Finally, we tested whether the effect of secure-base leadership on taking-charge behavior is weakened or disappears under the premise of considering the effect of psychological availability in order to verify whether the mediating utility of psychological availability exists. As shown in Table 4, after controlling demographic variables, M4 shows that secure-base leadership has a significant positive impact on taking-charge behavior (β = 0.300, *p* < 0.001); therefore, Hypothesis 1 is validated. M2 indicates that secure-base leadership has a significant positive impact on psychological availability (β = 0.274, *p* < 0.001), and thus Hypothesis 2 is validated. According to M4 and M5, after adding psychological availability to M4, a significant positive correlation exists between psychological availability and taking-charge behavior (β = 0.784, *p* < 0.001). With the addition of psychological availability, secure-base leadership still shows a significant positive impact on taking-charge behavior (β = 0.085, *p* < 0.05), but it is significantly reduced, indicating that psychological availability plays a mediating role in the positive and significant relationship between secure-base leadership and taking-charge behavior. Hypothesis 3 thus is validated.

M4 displays the results of the mediation effect of psychological availability in the relationship between secure-base leadership and employee’ taking-charge behavior. A sample size of 5000 and a 95% confidence interval with Process 4.1 were set, as shown in Table 5. The results showed that after controlling the demographics variables, the direct effect size of secure-base leadership on employee’ taking-charge behavior was 0.094, the confidence interval was [0.016, 0.178], excluding 0, and the direct effect was significant. In the path of secure-base leadership influencing employees’ taking-charge behavior, the effect size of the mediation effect of psychological availability is 0.238, accounting for 71.69% of the total effect, with a confidence interval of [0.114, 0.363], excluding 0, which further indicates that the mediating effect of psychological availability is significant.

#### 4.4.2. Moderating Effect Test

This study took independent self-construal as a moderating variable and incorporated it between psychological availability and taking-charge behavior to test its impact on the path of psychological availability and taking-charge behavior. We used statistical software SPSS 27.0 and Process 4.1 plugins to test the moderating effect of independent self-construal. We set employee gender, age, education level, and tenure as control variables.

This study was based on the stepwise law of the return method described by Wen Zhonglin. After incorporating the control variables into the regression model, the psychological availability of the mediating variable was added to the regression equation, and independent self-construal was a moderating variable. The interaction term of psychological availability and independent self-construal centralization processing added. If the regression coefficient of the interaction term was significant or there was a significant difference among the three models, it indicated a significant moderating effect. The regression results are shown in Table 6. According to M9, the interaction term between psychological availability and independent self-construal significantly positively affects taking-charge behavior (β = 0.107, *p* < 0.01) and indicates that the higher employees’ independent self-construal, the stronger the impact of psychological availability on taking-charge behavior. Moreover, the R^2^ of M8 and M9 increased from 0.716 to 0.727, enhancing the explanatory power of the model. Therefore, it can be concluded that independent self-construal positively moderates the impact of psychological availability on taking-charge behavior. Hypothesis 4 is thus validated.

**Figure 2:**
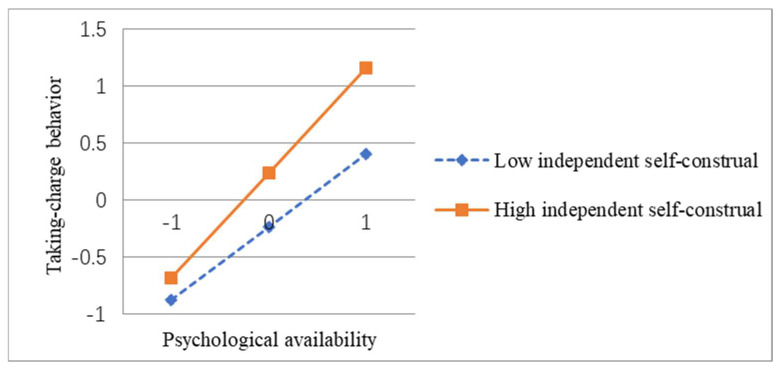
The moderating effect of independent self-construal.

To further visually demonstrate the moderating effect of independent self-construal, this study drew a moderating effect map based on Aiken’s method, as shown in Figure 2. It can be seen that after controlling the demographics variables, the slope of the line segment represented by the low independent self-construal (Mean − 1 SD) (β = 0.697, *p* < 0.001), the slope of the line represented by high independent self-construal (Mean + 1 SD) (β = 0.866, *p* < 0.001) is steeper, indicating that psychological availability has a more significant positive impact on taking-charge behavior under high independent self-construal. Hypothesis 4 is further supported.

#### 4.4.3. The Test of Moderated Mediation Effect

In order to further test the moderated mediating effect, we used Model 14 in the Process 4.1 plug-in, set the bootstrap to a 95% confidence interval, and repeated 5000 sampling tests. After controlling the demographics variables, the independent self-construal was tested for the moderated mediating effect, as shown in Table 7. From the table, it can be seen that under low independent self-construal, secure-base leaders have a significant indirect effect on taking-charge behavior through psychological availability (β = 0.202, 95% confidence interval = [0.096, 0.308], excluding 0), while under high independent self-construal, secure-base leadership has a significant indirect effect on taking-charge behavior through psychological availability (β = 0.254, 95% confidence interval = [0.119, 0.394], excluding 0). The difference between the high- and low-effect size of independent self-construal was 0.052, and the 95% confidence interval was [0.013, 0.105], excluding 0. The high- and low-effect size showed significant difference. Therefore, independent self-construal positively moderates the mediation effect of psychological availability in the relationship between secure-base leadership and taking-charge behavior. Hypothesis 5 is verified.

#### 4.4.4. Further Analysis

**Figure 3:**
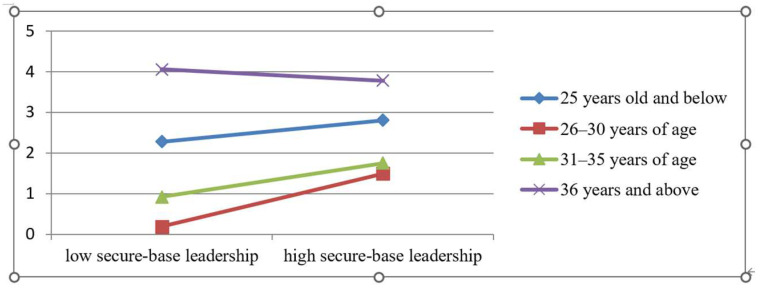
The moderating effect of age.

**Figure 4:**
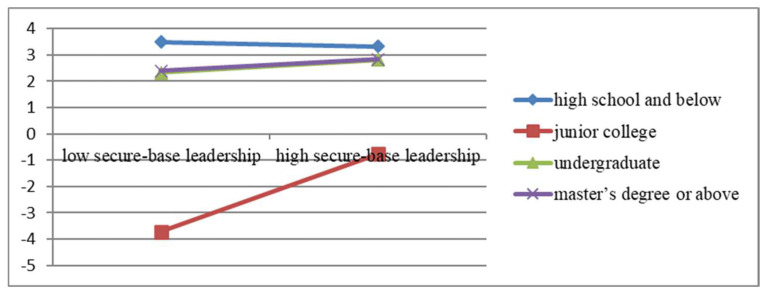
The moderating effect of education level.

The present study further tests the potential moderation effect of gender, age, education level, and tenure. The results indicate that the effect of gender (*p* = 0.1452) and tenure (*p* = 0.3181) did not reach statistical significance. Age significantly moderates the effect of secure-base leadership on taking-charge behavior (*p* = 0.0442, *p* < 0.05), which is vividly depicted in Figure 3. Among the four age groups, the slope represented by the 26–30 years of age is the highest, indicating that effect of secure-base leadership on such behavior achieves the optional. As shown in Figure 4, education level also has a significant outcome in their relationship (*p* = 0.000). For the education categories, the slope of the junior college possesses the highest value, suggesting employees attaining junior education have the highest possibility to take charge behaviors with the influence of secure-base leadership.

## 5. Discussion and Conclusions

The present study addresses whether and how secure-base leadership affects the employee taking-charge behaviors. According to the empirical analyses with the survey data of 250 enterprise employees, we found that (a) secure-base leadership has a significant positive effect on employees’ taking-charge behavior; (b) psychological availability plays a mediating role between secure-base leadership and employees’ taking-charge behavior; (c) independent self-construal plays a positive moderating role between psychological availability and employees’ taking-charge behavior; (d) independent self-construal positively moderates the mediating effects of psychological availability between secure-base leadership and employees’ taking-charge behavior, the higher the employee’s independent self-construal, the stronger the mediating role of psychological availability; (e) among the control variables, age and education level have a significant impact on the process of employees’ taking-charge behavior influenced by secure-base leadership.

### 5.1. Theoretical Discussions

This study enriches the theoretical research on secure-base leadership and expands the research on the outcome variables of such leadership. Since the concept of secure-base leadership is relatively new, research on the topic has been sparse. Existing studies are mainly based on attachment theory [20], self-determination theory [37], and conservation of resources theory [53], which all confirm that secure-base leadership has a positive effect on employees’ innovative, proactive, and communicative behavior. Recent research has explored the effects of secure-base leadership on employee creativity from the perspective of creative leadership theory [17]. There is also a lack of research on the impact of secure-base leadership on employees’ taking-charge behavior based on social cognitive theory. On the one hand, our study expands the research on the outcome variables of secure-base leadership, and at the same time, promotes the research on the motivating factors of employees’ taking-charge behavior, and enriches the mechanism of the role of secure-base leadership on employees’ taking-charge behavior. On the other hand, based on social cognitive theory to explore the influence of secure-base leadership on employees’ taking-charge behavior, it provides a more theoretical basis for the research of secure-base leadership.

The study also introduces psychological availability and explores its mediating role in the process of motivating employees’ taking-charge behavior in secure-base leadership. Based on social cognitive theory, this study takes secure-base leadership as a situational factor that conveys care to employees, encourages them to challenge the authority of the organization, stimulates their emotional resources on the basis of satisfying their physiological health needs, and enhances their psychological perception of a good organizational climate, which thus promotes employees’ taking-charge behavior. Psychological factors that have been studied regarding stimulating employees’ taking-charge behavior are mostly self-efficacy [4], psychological empowerment [6,54] and safety [55], and few scholars have taken psychological availability as mediator. This study enriches the mechanism of psychological availability as a mediator to stimulate employees’ taking-charge behavior. At the same time, this study verified that psychological availability is an influencing channel by which secure-base leadership stimulates employees’ taking-charge behavior, opening up the black box of how secure-base leadership shapes employees’ taking-charge behavior [17], enriching and extending the theoretical underpinnings regarding the outcomes of secure-base leadership [56], and providing theoretical evidence to literature on psychological availability [57] as the psychological motivation driving employees’ taking-charge behavior.

Moreover, this study incorporates the moderating variable of independent self-construal and expands the boundary effect on the relationship between psychological availability and employees’ taking-charge behavior. In recent years, the literature has regarded independent self-construal as the boundary condition on the outcomes of humble leadership behavior through conservation of resources theory [58] and supervisor bottom-line mentality through trait activation theory [46]. However, independent self-construal has not yet been used as a boundary condition to examine the outcome of secure-base leadership. Employees with high independent self-construal are more eager to make personal and organizational progress, want a degree of freedom at work, take the initiative to identify organizational problems and put forward insights that are beneficial to the organization, which amplifies the effect of psychological availability on taking-charge behavior, and further improves the role of their individual characteristics in the process of psychologically influencing behaviors from a cultural values perspective.

### 5.2. Practical Insights

First, the organization should focus on cultivating a secure-base leadership style. Organizations can adopt a practice of prioritizing the hiring of managers who possess secure-base leadership attributes such as availability, encouragement, and noninterference. At the same time, organizations should also establish sound communication channels, effective feedback systems, and targeted reward and punishment mechanisms to guide managers to adopt a secure-base leadership approach. Managers themselves should be good at observing the physical health and psychological changes of employees in their daily management process, and develop a humanized management model. For example, regularly carrying out company team building activities to enhance emotions among employees, and providing positive emotional support to employees while ensuring their physical health. In addition, managers should appropriately delegate certain powers, provide opportunities for employees to exercise themselves, develop their mental models of daring to take risks and innovate, motivate employees to engage in challenging work, foster confidence in employees to optimize organizational operation models, and actively change outdated rules, regulations, and operating procedures in existing organizations, for example, by providing employees with more opportunities for growth and development through learning, training, promotion, etc., and formulating career plans for employees.

Second, organizations should focus on improving employees’ psychological availability. Given that different employees have different needs, managers can respect their subordinates, accept their differences, enhance their psychological well-being, and thus improve their psychological availability [40]. One side, managers should always pay attention to the feedback needs of employees. Organizations can establish a good communication platform, accept suggestions widely, provide convenient channels for employees to express their ideas, improve their perception of their own status in the organization, and make employees truly feel that they are a part of the organization. On the other hand, managers should establish friendly relationships with employees, make them feel valued by leaders, strengthen their correct understanding of the good values of the organization, and strengthen their sense of identification with the organization. In addition, managers should try their best to help employees improve themselves, encourage them to actively learn, improve their work abilities, and create a fair, just, and open organizational atmosphere to encourage employees to demonstrate taking-charge behavior. For example, managers can learn from Gree Electric Chairman Dong Mingzhu’s attitude towards employees. Dong Mingzhu attaches great importance to employees’ mental health and work environment, raises wages for employees, buys houses for employees, pays attention to talent cultivation, and establishes a comprehensive talent system within Gree Electric that “selects, nurtures, uses, and retains”. At the same time, managers should propel employees to participate in physical exercise activities, organize competitions, and award prizes to those with excellent performance. In addition, it is necessary to cultivate employees’ ability to control their emotions, and provide training on interpersonal relationships, communication skills, problem solving, and adaptability. For example, establishing an employee psychological counseling room to capture the psychological dynamics of employees, provide psychological guidance, encourage them to release pressure, and adjust their mindset.

Third, organizations should be attentive to employees’ independent self-construal awareness. When the employee recruitment process, enterprises should focus on whether prospective employees actively pursue self-improvement, care about development of the team and individuals, and pose involve inquiries about industry prospects and personal plans during the interview process. Priority should be given to selecting employees with independent self-construal traits. In addition, managers should build a platform to showcase high independent and self-construal employees that highlights their dominant position. Managers should strive for employees to leverage their talents, achieving a match between individuals and positions. For example, enterprises can provide tailored training, classification management, and leaders’ demonstration to guide employees’ self-construal necessary for organizational development and enhance their resilience to potential uncertainty.

### 5.3. Research Limitations and Future Prospects

There are some deficiencies in the research data. The data for the four variables in this study were all concurrently completed, such that the cross-sectional data could not track the dynamic impact of secure-base leadership on employees’ taking-charge behavior. Therefore, in the future, the data collection method can be optimized by using situational experiments or increasing the time interval of data collection.

In addition, this study only focuses on the impact of secure-base leadership on employees’ taking-charge behavior. In the future, further research can be conducted on the impact of secure-base leadership on other behaviors such as employee- or team-deviant innovation.

The research perspective is relatively singular. This study has only explored the mechanism of the influence of secure-base leadership on employees’ taking-charge behavior from the social-cognitive theory perspective and introduces the mediating variable of psychological availability from the perspective of the social-cognitive theory. In the future, we can explore the influence mechanism of secure-base leadership on employee behaviors from the conservation of resources theory, leader–member exchange theory and the social information processing theory.

## Figures and Tables

**Table 1:** Respondents’ demographics.

Variables	Categories	Frequency (%)
Gender	Male	52.8
	Female	47.2
Age	25 years old and below	71.2
	26–30 years of age	20.8
	31–35 years of age	3.2
	36 years and above	4.8
Education level	High school and below	4.4
	Junior college	10.8
	Undergraduate	53.2
	Master’s degree or above	31.6
Tenure	One year and below	57.2
	2–5 years	31.6
	6–9 years	8
	10 years and above	3.2

**Table 2:** Results of the confirmatory factor analysis.

Measurement Models	χ^2^/df	IFI	CFI	TLI	RMSEA
Four-factor model (SBL, PA, ISC, TCB)	2.845	0.936	0.935	0.921	0.086
Three-factor model (SBL, PA + ISC, TCB)	10.232	0.669	0.666	0.604	0.193
Two-factor model (SBL, PA + ISC + TCB)	10.297	0.660	0.657	0.601	0.193
Single-factor model (SBL + PA + ISC + TCB)	11.886	0.597	0.595	0.533	0.209
Data standards	<5	>0.9	>0.9	>0.9	<0.1

Note: N = 250; SBL = secure-base leadership, PA = psychological availability, ISC = independent self-construal, TCB = taking-charge behavior. “+” = two factors merged into one.

**Table 3:** The mean, standard deviation, and correlation coefficient of the main variables.

Variables	Mean	SD	1	2	3	4	5	6	7	8
1. Gender	1.47	0.500	1							
2. Age	1.42	0.773	−0.001	1						
3. Education level	3.12	0.767	0.061	0.139 *	1					
4. Tenure	1.57	0.774	−0.047	0.628 **	−0.346 **	1				
5. Secure-base leadership	3.476	0.671	−0.040	−0.021	−0.157 *	0.049	1			
6. Taking-charge behavior	3.376	0.743	−0.205 **	−0.128 *	−0.011	−0.021	0.300 **	1		
7. Psychological availability	3.571	0.722	−0.102	−0.107	0.060	−0.031	0.260 **	0.821 **	1	
8. Independent self-construal	3.677	0.600	−0.145 *	−0.094	−0.132 *	0.054	0.352 **	0.435 **	0.331 **	1

Note: N = 250, ** *p* < 0.01, * *p* < 0.05.

**Table 4:** Test results for direct and mediating effects.

Variables and Models	Psychological Availability		Taking-Charge Behavior		
	M1	M2	M3	M4	M5
Gender	−0.104	−0.096	−0.204 **	−0.195 **	−0.120 ***
Age	−0.230 *	−0.233 **	−0.227 *	−0.230 **	−0.048
Education level	0.154 *	0.199 **	0.082	0.130	−0.026
Tenure	0.162	0.166	0.140	0.145	0.015
Secure-base leadership		0.274 ***		0.300 ***	0.085 *
Psychological availability					0.784 ***
R^2^	0.039	0.113	0.067	0.155	0.700
ΔR^2^		0.073		0.088	0.545
ΔF	2.516 *	20.155 ***	4.405 **	25.341 ***	440.978 ***

Note: N = 250, *** *p* < 0.001, ** *p* < 0.01, * *p* < 0.05.

**Table 5:** Breakdown of total effect, direct effect, and mediating effect.

Effect Type	Effect	Boot SE	Bootstrap 95%CI		Relative Effect Proportion
			Boot LLCI	Boot ULCI	
Total effect	0.332	0.085	0.167	0.503	100%
Direct effect	0.094	0.041	0.016	0.178	28.31%
Indirect effect	0.238	0.063	0.114	0.363	71.69%

**Table 6:** Test results of moderating effect.

Variables and Models	Taking-Charge Behavior			
	M6	M7	M8	M9
Gender	−0.204 **	−0.120 ***	−0.103 **	−0.095 **
Age	−0.227 *	−0.042	−0.025	−0.017
Education level	0.082	−0.043	−0.026	−0.017
Tenure	0.140	0.010	−0.005	−0.004
Psychological availability		0.807 ***	0.755 ***	0.759 ***
Independent self-construal			0.165 ***	0.191 ***
Psychological availability × Independent self-construal				0.107 **
R^2^	0.067	0.693	0.716	0.727
ΔR^2^		0.626	0.023	0.011
ΔF	4.405 **	497.972 ***	19.791 ***	9.375 **

Note: N = 250, *** *p* < 0.001, ** *p* < 0.01, * *p* < 0.05.

**Table 7:** Mediating effect test of psychological availability under the independent self-construal level.

Moderator Variable Levels	Effect	Boot SE	Boot LLCI	Boot ULCI
Low independent self-construal	0.202	0.054	0.096	0.308
High independent self-construal	0.254	0.070	0.119	0.394
Discrepancy (difference between high and low)	0.052	0.023	0.013	0.105

## Data Availability

The data employed in this study are available from the corresponding author.
